# Safety and Effectiveness of a Catheter With Contact Force and 6-Hole Irrigation for Ablation of Persistent Atrial Fibrillation in Routine Clinical Practice

**DOI:** 10.1001/jamanetworkopen.2022.27134

**Published:** 2022-08-17

**Authors:** Sanket S. Dhruva, Shumin Zhang, Jiajing Chen, Peter A. Noseworthy, Amit A. Doshi, Kolade M. Agboola, Jeph Herrin, Guoqian Jiang, Yue Yu, Guy Cafri, Kimberly Collison Farr, Keondae R. Ervin, Joseph S. Ross, Paul M. Coplan, Joseph P. Drozda

**Affiliations:** 1 Section of Cardiology, Department of Medicine, University of California, San Francisco School of Medicine, San Francisco; 2 San Francisco Veterans Affairs Medical Center, San Francisco, California; 3 MedTech Epidemiology and Real-World Data Sciences, Office of the Chief Medical Officer, Johnson & Johnson, New Brunswick, New Jersey; 4 Mercy Research, Mercy Health, Chesterfield, Missouri; 5 Department of Cardiovascular Medicine, Mayo Clinic, Rochester, Minnesota; 6 Mercy Clinic, Mercy Health, St Louis, Missouri; 7 Section of Cardiovascular Medicine, Department of Internal Medicine, Yale School of Medicine, New Haven, Connecticut; 8 Department of Artificial Intelligence and Informatics, Mayo Clinic, Rochester, Minnesota; 9 Department of Quantitative Health Sciences, Mayo Clinic, Rochester, Minnesota; 10 National Evaluation System for Health Technology Coordinating Center, Medical Device Innovation Consortium, Arlington, Virginia; 11 Department of Internal Medicine, Yale School of Medicine, New Haven, Connecticut; 12 Center for Outcomes Research and Evaluation, Yale–New Haven Hospital, New Haven, Connecticut

## Abstract

**Importance:**

The ThermoCool SmartTouch catheter (ablation catheter with contact force and 6-hole irrigation [CF-I6]) is approved by the US Food and Drug Administration (FDA) for paroxysmal atrial fibrillation (AF) ablation and used in routine clinical practice for persistent AF ablation, although clinical outcomes for this indication are unknown. There is a need to understand whether data from routine clinical practice can be used to conduct regulatory-grade evaluations and support label expansions.

**Objective:**

To use health system data to compare the safety and effectiveness of the CF-I6 catheter for persistent AF ablation with the ThermoCool SmartTouch SurroundFlow catheter (ablation catheter with contact force and 56-hole irrigation [CF-I56]), which is approved by the FDA for this indication.

**Design, Setting, and Participants:**

This retrospective, comparative-effectiveness cohort study included patients undergoing catheter ablation for persistent AF at Mercy Health or Mayo Clinic from January 1, 2014, to April 30, 2021, with up to a 1-year follow-up using electronic health record data.

**Exposures:**

Use of the CF-I6 or CF-I56 catheter.

**Main Outcomes and Measures:**

The primary safety outcome was a composite of death, thromboembolic events, and procedural complications within 7 to 90 days. The exploratory effectiveness outcome was a composite of AF-related hospitalization events after a 90-day blanking period. Propensity score weighting was used to balance baseline covariates. Risk differences were estimated between catheter groups and averaged across the 2 health care systems, testing for noninferiority of the CF-I6 vs the CF-I56 catheter with respect to the safety outcome using 2-sided 90% CIs.

**Results:**

Overall, 1450 patients (1034 [71.3%] male; 1397 [96.3%] White) underwent catheter ablation for persistent AF, including 949 at Mercy Health (186 CF-I6 and 763 CF-I56; mean [SD] age, 64.9 [9.2] years) and 501 at Mayo Clinic (337 CF-I6 and 164 CF-I56; mean [SD] age, 63.7 [9.5] years). A total of 798 (55.0%) had been treated with class I or III antiarrhythmic drugs before ablation. The safety outcome (CF-I6 – CF-I56) was similar at both Mercy Health (1.3%; 90% CI, −2.1% to 4.6%) and Mayo Clinic (−3.8%; 90% CI, −11.4% to 3.7%); the mean difference was noninferior, with a mean of 0.5% (90% CI, −2.6% to 3.5%; *P* < .001). The effectiveness was similar at 12 months between the 2 catheter groups (mean risk difference, −1.8%; 90% CI, −7.3% to 3.7%).

**Conclusions and Relevance:**

In this cohort study, the CF-I6 catheter met the prespecified noninferiority safety criterion for persistent AF ablation compared with the CF-I56 catheter, and effectiveness was similar. This study demonstrates the ability of electronic health care system data to enable safety and effectiveness evaluations of medical devices.

## Key Points

**Question:**

Can data from routine clinical practice be used to evaluate the safety and effectiveness of a device, using as an exemplar a catheter with contact force and 6-hole irrigation for persistent atrial fibrillation ablation?

**Findings:**

In this cohort study of 1450 patients receiving catheter ablation at 2 health care systems, a catheter with contact force and 6-hole irrigation had similar safety and effectiveness for persistent atrial fibrillation ablation to a catheter with contact force and 56-hole irrigation (which has a US Food and Drug Administration–approved indication for persistent atrial fibrillation).

**Meaning:**

This study suggests that health system data are useful in postmarket evaluation of cardiovascular device safety and effectiveness.

## Teaser

This cohort study uses electronic health record data to compare the safety and effectiveness of a smart touch catheter with that of a smart touch surround flow catheter for persistent atrial fibrillation ablation in routine clinical practice.

## Introduction

Atrial fibrillation (AF) is the most common sustained heart rhythm disorder and is associated with adverse cardiovascular outcomes.^1^ A common subtype is persistent AF, defined as AF without interruption for at least 7 days.^2^ Randomized clinical trials have demonstrated clinical benefits with ablation compared with long-term medical therapy among patients with persistent AF.^3,4^ The 2014 American Heart Association/American College of Cardiology/Heart Rhythm Society AF guideline indicates that catheter ablation is reasonable for some patients with symptomatic persistent AF, and approximately 30% of AF ablations are performed for this indication.^5^

A radiofrequency catheter with contact force and 56-hole irrigation (CF-I56) (ThermoCool SmartTouch SurroundFlow, Biosense Webster Inc) has a Food and Drug Administration (FDA)–approved indication for treatment of persistent AF; this approval was received after a traditional investigational device exemption clinical trial.^6^ A radiofrequency catheter with contact force and 6-hole irrigation (CF-I6) (ThermoCool SmartTouch, Biosense Webster Inc) has an FDA-approved indication for paroxysmal AF; it is also used clinically for ablation of persistent AF. However, the short- and long-term clinical outcomes when the CF-I6 catheter is used for persistent AF ablation are unknown.

Use of the CF-I6 catheter for persistent AF in routine clinical practice provides an opportunity to compare safety and effectiveness outcomes with those of the CF-I56 catheter.^6^ There is increasing interest in leveraging what the FDA refers to as real-world data, including electronic health record (EHR) data, to generate FDA-termed real-world (hereafter referred to as data collected from routine clinical practice) evidence for evaluations of medical device safety and effectiveness. The 21st Century Cures Act of 2016^7^ emphasizes the importance of developing evidence from routine clinical practice for medical product evaluations. In 2017, the FDA released guidance stating that evidence from routine clinical practice has potential to support label expansions for medical devices.^8^ Several studies^9,10,11,12,13^ have used multiple sources, including data embedded within EHRs and registries, for medical device safety evaluations.

However, EHRs have never served as the sole data source for label expansion of a premarket approval device. Previous studies^14,15^ demonstrated the feasibility of using EHR data for a label expansion study of ablation catheters. Accordingly, in this study, we used EHR data to compare the safety and effectiveness of the CF-I6 catheter with the CF-I56 catheter among patients receiving persistent AF ablation in routine clinical practice. The goal was to inform a label extension submission to the FDA for the CF-I6 catheter to add persistent AF as a labeled indication by comparing it with the CF-I56 catheter.

## Methods

### Overall Study Design

This cohort study is a test case of the National Evaluation System for Health Technology Coordinating Center (NESTcc), an initiative focused on developing evidence from routine clinical practice about medical device safety and effectiveness to inform regulatory and clinical decision-making. The study protocol and statistical analysis plan were developed by the collaborators, approved by NESTcc, and updated based on FDA feedback through 2 Q-submission meetings^16^ and 2 sets of written comments. This is a 2-arm retrospective study that compares the CF-I6 and CF-I56 catheters. This study was deemed exempt from institutional review board review at Mercy Health and deemed minimal-risk research with a waiver of informed consent at Mayo Clinic. Both the protocol and statistical analysis plan were posted publicly on the European Network of Centres in Pharmacoepidemiology and Pharmacovigilance postauthorization studies register^17^ before hypothesis testing. This report follows the Strengthening the Reporting of Observational Studies in Epidemiology (STROBE) reporting guideline.

### Data Sources and Distributed Analytic Model

Data were obtained from the health information technology systems at Mercy Health and Mayo Clinic, including device data from supply chain systems and clinical data from EHR systems for January 1, 2014, to April 30, 2021. Mercy Health is a health system that operates in 4 states, and Mayo Clinic is a health system that operates in 5 states.

All analyses were conducted using a distributed analysis model in which each health care system analyzed its patient data using a standardized approach. Each health care system adopted the Observational Health Data Sciences and Informatics Observational Medical Outcomes Partnership (OMOP) common data model, version 5.3.1 for EHR data system management. This program allowed for standardized definition, capture, and integration of all study data elements.

### Inclusion and Exclusion Criteria

We included patients aged 18 years or older with at least 6 months of encounter history undergoing their first ablation for persistent AF by requiring a *Current Procedural Terminology* (*CPT*) or *International Statistical Classification of Diseases and Related Health Problems, Tenth Revision (ICD-10)* Procedure Coding System code for AF ablation or destruction of the right or left pulmonary veins (*CPT* code 93656 or *ICD-10* Procedure Coding System codes 025S3ZZ or 025T3ZZ, respectively) using the CF-I6 or CF-I56 catheter. Patients with a specific catheter exposure were identified using the unique device identifier in supply chain data linked to the EHR.^15^ We also required patients to have at least 1 of 3 *International Statistical Classification of Diseases, Tenth Revision, Clinical Modification* (*ICD-10-CM*) codes for persistent AF (I48.1, I48.11, or I48.19) in any diagnosis position associated with the ablation procedure encounter. We excluded patients who received ablation with both catheters during the index procedure, prior ablation, concomitant left atrial appendage occlusion ablation, concomitant atrioventricular node ablation (which we presumed was caused by incorrect coding and was identified during cohort construction), and prior heart transplant or long-term heart-assist system implantation.

### Outcomes

The primary safety outcome was a composite similar to that used in the clinical trial^18^ that supported label extension for the CF-I56 catheter. The FDA indicated that the primary objective should be safety because differences between the CF-I6 and CF-I56 catheters were not expected to affect device effectiveness, whereas improved cooling irrigation technology of the catheter tip with CF-I56 could lead to better safety outcomes than with the CF-I6 catheter. The safety outcome was a composite of death, acute myocardial infarction, acute stroke, transient ischemic attack, thromboembolism, diaphragmatic paralysis, pneumothorax, heart block, pulmonary edema, pericarditis, major vascular access complication, or bleeding that required transfusion within 7 days after the procedure; cardiac perforation or tamponade within 30 days; and pulmonary vein stenosis or atrioesophageal fistula within 90 days. Patients who may have had any components of this composite end point were identified by physician medical record review after use of an algorithm intended to be sensitive for identification of any patients who may have had any adverse event.

An effectiveness end point was examined as an exploratory outcome given anticipated clinical interest in the findings. The effectiveness outcome was a composite incidence of the following at 6 months and 1 year: rehospitalization for atrial tachyarrhythmia (including AF, atrial tachycardia [AT], and atypical atrial flutter [AFL]), rehospitalization for heart failure, electrical cardioversion for AF, AT, or AFL, or repeat ablation for AF, AT, or AFL. Consistent with guidelines^19^ and common practice,^18,20,21,22^ these outcomes were ascertained after a 90-day blanking period. Algorithms to identify these effectiveness outcomes were defined using *International Classification of Diseases, Ninth Revision* (*ICD-9*), *ICD-10*, or *CPT* codes validated in a nonanalytic data set. Effectiveness end points were only ascertained among patients followed up within the health system for up to 1 year, as evidenced by encounter records.^14^

### Physician-Led Safety Event Identification

Medical records for physician review were identified to ensure no safety events were missed using 3 criteria. First, we included the medical record of any patient with a hospitalization, readmission, or emergency department visit for any cause within 7 days after index ablation. Second, we included the medical record of any patient with a length of stay of 48 hours or longer after index ablation. Because patients are nearly always discharged within 48 hours after uncomplicated ablations, prolonged hospitalizations could signal a complication. Third, we included the medical record of any patient with a diagnosis or procedure code for any event in the composite safety end point that would not be expected to always lead to a hospitalization, readmission, or emergency department visit or length of stay of 48 hours or longer. For the 3 safety end points ascertained at more than 7 days after ablation, we used diagnosis and/or procedure codes indicative of these complications (derived, when possible, from the peer-reviewed literature^23^) to identify medical records for physician review. Medical record reviews were conducted by a total of 4 physicians (including P.A.N., A.A.D., and K.M.A.), 2 at each site. Three of these 4 physicians were cardiac electrophysiologists. The physicians were not blinded to the study hypothesis or catheter used. All safety event identification was conducted using a standardized data abstraction instrument with explicitly defined criteria. Interrater agreement was assessed across 10 medical records at each site. In the 2 cases of disagreement at Mercy Health, consensus was achieved through discussion between the 2 reviewing physicians. No physician reviewed a medical record for a patient in whom the physician had performed an ablation.

### Covariates

Covariates were patient demographic variables; patient medical history variables based on all available information before the index procedure; most current patient body mass index measurements before the index procedure; medications within 6 months before the index procedure; hospital and provider characteristics, including number of hospital beds and operator volume at the time of index ablation procedure; and ablation year (Table 1). We included race as a demographic variable, as there may be differences in clinical outcomes among patients with atrial fibrillation associated with race.

**Table 1.** Baseline Characteristics of Patients Treated with Catheter Ablation for Persistent Atrial Fibrillation, Stratified by Health Care System

Characteristic	Mercy Health			Mayo Clinic		
	No. (%)		*P* value	No. (%)		*P* value
	CF-I6 catheter (n = 186)	CF-I56 catheter (n = 763)		CF-I6 catheter (n = 337)	CF-I56 catheter (n = 164)	
Patient demographic characteristics						
Age, y						
<65	75 (40.3)	349 (45.7)	.41	186 (55.2)	68 (41.5)	.02
65-74	83 (44.6)	310 (40.6)		114 (33.8)	71 (43.3)	
≥75	28 (15.1)	104 (13.6)		37 (11.0)	25 (15.2)	
Sex						
Male	128 (68.8)	544 (71.3)	.56	244 (72.4)	118 (72.0)	>.99
Female	58 (31.2)	219 (28.7)		93 (27.6)	46 (28.0)	
Race or ethnicity						
White	177 (95.2)	732 (95.9)	.60	326 (96.7)	162 (98.8)	.29
Other^a^	9 (4.8)	31 (4.1)		11 (3.3)	2 (1.2)	
Medical history and arrhythmia-related information						
Elixhauser Comorbidity Index score						
≤3	50 (26.9)	183 (24.0)	.47	121 (35.9)	39 (23.8)	.009
>3	136 (73.1)	580 (76.0)		216 (64.1)	125 (76.2)	
BMI, mean (SD)^b^	31.8 (5.8)	32.8 (6.7)	.04	32.3 (6.4)	32.9 (7.8)	.38
PCI	13 (7.0)	49 (6.4)	.91	7 (2.1)	4 (2.4)	>.99
CABG	13 (7.0)	67 (8.8)	.52	7 (2.1)	4 (2.4)	>.99
Hypertension	147 (79.0)	615 (80.6)	.70	199 (59.1)	116 (70.7)	.01
Diabetes	38 (20.4)	198 (26.0)	.14	47 (13.9)	37 (22.6)	.02
Obstructive sleep apnea	86 (46.2)	364 (47.7)	.78	136 (40.4)	75 (45.7)	.30
Congestive heart failure	77 (41.4)	318 (41.7)	>.99	138 (40.9)	84 (51.2)	.04
Chronic pulmonary disease	52 (28.0)	215 (28.2)	>.99	73 (21.7)	48 (29.3)	.08
Stroke or transient ischemic attack or thromboembolism	20 (10.8)	96 (12.6)	.58	28 (8.3)	23 (14.0)	.07
Chronic renal disease	29 (15.6)	127 (16.6)	.81	19 (5.6)	23 (14.0)	.003
Anemia	12 (6.5)	62 (8.1)	.54	21 (6.2)	14 (8.5)	.44
Valve replacement	8 (4.3)	27 (3.5)	.78	14 (4.2)	6 (3.7)	.98
Mitral valve stenosis	3 (1.6)	5 (0.7)	.40	5 (1.5)	1 (0.6)	.68
Vascular disease^c^	58 (31.2)	274 (35.9)	.26	178 (52.8)	64 (39.0)	.005
Hospitalizations: atrial fibrillation related^d^	89 (47.8)	315 (41.3)	.12	131 (38.9)	86 (52.4)	.005
Electrical cardioversion for atrial fibrillation	136 (73.1)	543 (71.2)	.66	139 (41.2)	111 (67.7)	<.001
History of supraventricular arrythmia	89 (47.8)	352 (46.1)	.73	188 (55.8)	105 (64.0)	.10
History of ventricular arrhythmia	17 (9.1)	54 (7.1)	.42	38 (11.3)	24 (14.6)	.35
Implantable cardioverter-defibrillator or pacemaker	18 (9.7)	61 (8.0)	.55	23 (6.8)	15 (9.1)	.46
Medications within 6 mo before index ablation						
Antiarrhythmic drugs						
Class I or III	112 (60.2)	438 (57.4)	.54	155 (46.0)	93 (56.7)	.03
Class II or IV	172 (92.5)	697 (91.3)	.73	274 (81.3)	136 (82.9)	.75
Anticoagulant	167 (89.8)	681 (89.3)	.94	205 (60.8)	126 (76.8)	<.001
Antiplatelet	155 (83.3)	618 (81.0)	.53	88 (26.1)	46 (28.0)	.72
Operator and procedural characteristics						
Ablation year						
2014-2018	174 (93.5)	184 (24.1)	<.001	183 (54.3)	42 (25.6)	<.001
2019-2021	12 (6.5)	579 (75.9)		154 (45.7)	122 (74.4)	
Operator volume of atrial fibrillation ablations in the 12 mo before the index procedure^e^						
<25	9 (4.9)	247 (33.1)	<.001	95 (28.2)	23 (14.0)	<.001
25-50	18 (9.9)	49 (6.6)		198 (58.8)	135 (82.3)	
>50	155 (85.2)	450 (60.3)		44 (13.1)	6 (3.7)	
Missing	4 (2.2)	17 (2.2)		0	0	
Hospital size, No. of beds						
≥500	186 (100)	763 (100)	NA	301 (89.3)	10 (6.1)	<.001
<500	0	0		36 (10.7)	154 (93.9)	

Abbreviations: BMI, body mass index (calculated as weight in kilograms divided by height in meters squared); CABG, coronary artery bypass grafting; CF-I6, catheter with contact force and 6-hole irrigation; CF-I56, catheter with contact force and 56-hole irrigation; NA, not applicable; PCI, percutaneous coronary intervention.

^a^ Other refers to American Indian or Alaska Native, Asian, Black or African American, Hispanic or Latino, Native Hawaiian or Other Pacific Islander, other, and unknown.

^b^ Data are missing for 11 patients in the CF-I6 group and 1 patient in the CF-I56 catheter group at the Mayo Clinic.

^c^ Defined by prior myocardial infarction, peripheral arterial disease, or aortic plaque.

^d^ Defined as a hospitalization in which the primary discharge diagnosis was atrial fibrillation.

^e^ Percentages were based on data with known values.

### Statistical Analysis

We used a noninferiority design with a prespecified noninferiority margin of δ = 0.07 for comparison of the CF-I6 and CF-I56 catheters for the primary composite safety end point. The noninferiority margin was derived from the incidence of the composite safety end point from prior publications.^18,24,25,26^ Data were standardized in the OMOP common data model to facilitate common analyses at each health care system. Analyses were performed separately in each data set through standard queries, allowing each health care system to maintain patient EHR data within their organization. Demographic, clinical, procedural, and hospital and clinician characteristics at baseline were summarized descriptively for each group, with comparisons between the CF-I6 and CF-I56 catheters at each site using the χ^2^ test for categorical variables, 2-tailed, unpaired *t* test for continuous variables, and proportion test for all other variables that had 2 observed proportions. A 2-sided *P* < .05 was considered to be statistically significant.

Using a method described by FDA statisticians,^27^ we used 2 distinct analytic phases: covariate balancing blinded to study outcome data followed by hypothesis testing. In the first stage, 1 individual at each health care system created the analytical data set, removing any outcome data. These data were then provided to a statistician who was independent of the researchers at Mercy Health, Mayo Clinic, or Johnson & Johnson. This statistician built separate propensity score models for data from each health care system. These logistic regression models used the catheter group status as the outcome and all baseline characteristics (Table 1) as covariates. All covariates were prespecified in the study protocol and selected based on the literature,^18,25^ study cardiologist subject matter expertise, and FDA input. All prespecified covariates were included in the model unless an attribute represented by a covariate was absent in 1 treatment group. The propensity score was the estimated probability of receiving ablation with the CF-I6 catheter.

Covariate balance was examined under both propensity score stratification and average treatment effect on the treated weighting.^28^ The approach with the best covariate balance was selected.^29^ Propensity score average treatment effect on the treated weighting with trimming of weights at the 95th percentile gave the best balance of covariates. In the second stage, data balanced on baseline covariates were then linked back to outcomes and analyzed at each health care system; because of our distributed analytic approach, results were not combined. Results were subsequently pooled across health care systems using inverse variance weighting.

For the primary composite safety end point, survival analyses were conducted to estimate the risk difference between the 2 catheter groups. Risk in each catheter group was calculated as (1 –survival estimate) at 90 days. The conventional Kaplan-Meier estimator was used to estimate survival in the CF-I6 group with the Greenwood estimate of the variance. A weighted Kaplan-Meier estimator^30^ was used to calculate survival in the CF-I56 group using an infinitesimal jackknife estimate of the variance.^31^ The variance of the risk difference is the sum of the variances in these 2 groups. Risk differences and their variances were calculated in each health care system and subsequently averaged with a fixed-effects model using inverse variance weights. The upper bound of the 2-sided 90% CI for the mean risk difference was compared with the prespecified noninferiority margin of δ = 0.07 for hypothesis testing. A 2-sided 90% CI (equivalent to a 1-sided 95% CI) was used because the research hypothesis was that the CF-I6 (older) catheter was as safe as or had no worse safety than the CF-I56 (newer) catheter that has FDA approval for the persistent AF indication and not that it had better safety.

For the exploratory effectiveness end point, the same methods were applied as when performing the safety analyses, except the period for outcome assessment varied (6 months or 1 year, with a 90-day blanking-period) and no hypothesis testing was performed; instead, we report 90% CIs to compare rates of the effectiveness end points between the CF-I6 and CF-I56 cohorts.

We also conducted 2 sensitivity analyses for the primary composite safety outcome on covariate-balanced data. Within Mayo Clinic’s data, we removed hospital bed size as a balancing variable because all patients at Mercy Health received an ablation at a hospital with at least 500 beds. We also examined safety outcomes among those who had received a Class I or III antiarrhythmic medication within 6 months before the index ablation.

Covariate weighting analyses were performed using Stata software, version 17 (StataCorp LLC). Hypothesis testing analyses were conducted using R Studio, version 1.4 (R Foundation for Statistical Computing).

## Results

### Baseline Characteristics

In total, 1450 patients (1034 [71.3%] male; 1397 [96.3%] White) underwent catheter ablation for persistent AF, including 949 at Mercy Health (186 CF-I6 and 763 CF-I56; mean [SD] age, 64.9 [9.2] years) and 501 at Mayo Clinic (337 CF-I6 and 164 CF-I56; mean [SD] age, 63.7 [9.5] years) (Table 1). A total of 617 (42.6%) had a history of congestive heart failure, and 574 (39.6%) a history of vascular disease. A total of 798 (55.0%) had been treated with class I or III antiarrhythmic drugs, and 621 (42.8%) had a history of AF-related hospitalization. Among patients who met the inclusion criteria, 365-day follow-up data were available for 173 of 186 patients (93.0%) treated with the CF-I6 catheter and 464 of 763 patients (60.8%) treated with the CF-I56 catheter at Mercy Health; at Mayo Clinic, 365-day follow-up data were available for 240 of 337 patients (71.2%) treated with the CF-I6 catheter and 101 of 164 patients (61.6%) treated with the CF-I56 catheter (eTable 1 in the Supplement). Loss to follow-up was primarily attributable to administrative censoring; because the CF-I56 was a newer catheter, it had more administrative censoring.

### Covariate Balance

Before covariate balancing, 2 covariates at Mercy Health and 14 covariates at Mayo Clinic had absolute standardized differences greater than 0.20 (eTables 2 and 3 in the Supplement). The optimal covariate balance at both Mercy Health and Mayo Clinic was achieved with weights trimmed at the 95th percentile.

### Primary Safety Outcome Results

Overall, medical records of 77 patients (8.1%) at Mercy Health and 158 (31.5%) at Mayo Clinic were reviewed by physicians based on the 3 criteria described above (eTable 4 in the Supplement). This review identified 63 safety events in 61 patients across the 2 health systems (Table 2). In total, 8 patients (4.3%) in the CF-I6 group at Mercy Health and 21 (6.2%) at Mayo Clinic had safety events within the prespecified periods; among patients treated with the CF-I56 catheter, 22 (2.9%) at Mercy Health and 10 (6.1%) at Mayo Clinic had safety events within the prespecified periods (Table 2). The most common adverse events were pericarditis and pulmonary edema. There were no deaths.

**Table 2.** Safety Events Included in the Composite Safety End Point by Ablation Catheter Used at Both Health Care Systems (Covariate-Unadjusted Data)^a^

Event	No. (%) of patients or events			
	Mercy Health		Mayo Clinic	
	CF-I6 catheter (n = 186)	CF-I56 catheter (n = 763)	CF-I6 catheter (n = 337)	CF-I56 catheter (n = 164)
Within 7 d of index ablation				
Death	0	0	0	0
Acute myocardial infarction	0	0	0	0
Acute stroke or cerebrovascular accident	1 (0.5)	1 (0.1)	0	0
Transient ischemic attack	0	0	0	0
Thromboembolism	0	0	0	0
Heart block	0	2 (0.3)	0	0
Pericarditis	2 (1.1)	6 (0.8)	21 (6.2)	10 (6.1)
Diaphragmatic paralysis	0	0	0	0
Pneumothorax	0	0	0	0
Pulmonary edema	3 (1.6)	9 (1.2)	0	0
Major vascular access complication or bleeding requiring transfusion	2 (1.1)	4 (0.5)	0	0
Within 30 d of index ablation				
Cardiac tamponade or perforation	0	2 (0.3)	0	0
Within 90 d of index ablation				
Pulmonary vein stenosis	0	0	0	0
Atrioesophageal fistula	0	0	0	0
No. of safety events within prespecified periods^b^	8 (4.3)	24 (3.1)	21 (6.2)	10 (6.1)
No. of patients who had a safety event within prespecified time	8 (4.3)	22 (2.9)	21 (6.2)	10 (6.1)

Abbreviations: CF-I6, catheter with contact force and 6-hole irrigation; CF-I56, catheter with contact force and 56-hole irrigation.

^a^ Percentages are simple proportions without accounting for censoring.

^b^ Duplicate patients may be included because of multiple events in a patient.

After covariate balancing, the risk difference comparing patients treated with the CF-I6 vs CF-I56 catheter was 1.3% (90% CI, −2.1% to 4.6%) at Mercy and −3.8% (90% CI, −11.4% to 3.7%) at Mayo Clinic (Table 3; eFigures 1 and 2 in the Supplement). Combining results from both health systems, the mean risk difference was 0.5% (90% CI, −2.6% to 3.5%). The upper bound of the CI of 3.5% was lower than prespecified noninferiority margin of 7% (*P* < .001 for noninferiority).

**Table 3.** Cumulative Incidences and Risk Differences of Composite Safety Outcome Using Covariate-Unadjusted Data and Propensity Score–Balanced Data From Both Health Care Systems^a^

Catheter group	Cumulative incidence, % (90% CI)
**Unadjusted data**	
Mercy Health	
CF-I6 catheter	4.4 (1.9 to 6.9)
CF-I56 catheter	2.9 (1.9 to 3.9)
Difference	1.5 (−1.3 to 4.3)
Mayo Clinic	
CF-I6 catheter	6.2 (4.0 to 8.4)
CF-I56 catheter	6.0 (3.0 to 9.1)
Difference	0.1 (−3.8 to 4.1)
**Covariate-balanced data**	
Mercy Health	
CF-I6 catheter	4.4 (1.9 to 6.9)
CF-I56 catheter	3.1 (1.0 to 5.2)
Difference	1.3 (−2.1 to 4.6)
Mayo Clinic	
CF-I6 catheter	6.2 (4.0 to 8.4)
CF-I56 catheter	10.1 (2.8 to 17.3)
Difference	−3.8 (−11.4 to 3.7)
Mean difference across Mercy Health and Mayo Clinic^b^	0.5 (−2.6 to 3.5)

Abbreviations: CF-I6, catheter with contact force and 6-hole irrigation; CF-I56, catheter with contact force and 56-hole irrigation.

^a^ Cumulative incidences were calculated from survival analyses.

^b^ *P* < .001 for the mean difference in cumulative incidence compared with a noninferiority margin of 0.07.

### Effectiveness Outcome Results

Within 12 months after ablation, of patients treated with the CF-I6 catheter, 26 (14.0%) at Mercy Health and 33 (9.8%) at Mayo Clinic had effectiveness events; among patients treated with the CF-I56 catheter, 112 (14.7%) at Mercy Health and 10 (6.1%) at Mayo Clinic had effectiveness events (Table 4). The most common event was rehospitalization for atrial tachyarrhythmias. After covariate balancing, the mean risk difference comparing patients treated with the CF-I6 vs CF-I56 catheters within 12 months across Mercy Health and Mayo Clinic was −1.8% (90% CI, −7.3% to 3.7%) (Table 5; eFigures 3 and 4 in the Supplement).

**Table 4.** Effectiveness Events by Ablation Catheter Used at Both Health Care Systems (Covariate-Unadjusted Data)

Event	No. (%) of patients or events			
	Mercy Health		Mayo Clinic	
	CF-I6 catheter (n = 186)	CF-I56 catheter (n = 763)	CF-I6 catheter (n = 337)	CF-I56 catheter (n = 164)
**Within 6 mo after ablation**				
Rehospitalization for atrial tachyarrhythmia	7 (3.8)	23 (3.0)	13 (3.9)	4 (2.4)
Rehospitalization for heart failure	0	6 (0.8)	2 (0.6)	0
Electrical cardioversion for atrial tachyarrhythmia	0	29 (3.8)	4 (1.2)	0
Repeated ablation for atrial tachyarrhythmia	6 (3.2)	13 (1.7)	10 (3.0)	3 (1.8)
No. of effectiveness events^a^	13 (7.0)	71 (9.3)	29 (8.6)	7 (4.3)
No. of patients who had an effectiveness event	9 (4.8)	58 (7.6)	15 (4.5)	4 (2.4)
**Within 1 y after ablation**				
Rehospitalization for atrial tachyarrhythmia	11 (5.9)	39 (5.1)	28 (8.3)	10 (6.1)
Rehospitalization for heart failure	1 (0.5)	12 (1.6)	3 (0.9)	1 (0.6)
Electrical cardioversion for atrial tachyarrhythmia	11 (5.9)	60 (7.9)	11 (3.3)	1 (0.6)
Repeated ablation for atrial tachyarrhythmia	9 (4.8)	20 (2.6)	18 (5.3)	6 (3.7)
No. of effectiveness events^a^	32 (17.2)	131 (17.2)	60 (17.8)	18 (11.0)
No. of patients who had an effectiveness event	26 (14.0)	112 (14.7)	33 (9.8)	10 (6.1)

Abbreviations: CF-I6, catheter with contact force and 6-hole irrigation; CF-I56, catheter with contact force and 56-hole irrigation.

^a^ Duplicate patients may be included because of multiple events in a patient.

**Table 5.** Cumulative Incidences and Risk Differences of Composite Effectiveness Outcome at 6 and 12 Months Using Propensity Score–Balanced Data From Both Health Care Systems

Catheter group	Cumulative incidence, % (90% CI)
**6 Months**	
Mercy Health	
CF-I6 catheter	5.0 (2.3 to 7.7)
CF-I56 catheter	9.0 (5.5 to 12.5)
Difference	−4.0 (−8.5 to 0.5)
Mayo Clinic	
CF-I6 catheter	5.0 (2.9 to 7.1)
CF-I56 catheter	0.4 (0.0 to 0.9)
Difference	4.5 (2.3 to 6.8)
Mean difference across both Mercy Health and Mayo Clinic	2.8 (0.8 to 4.8)
**12 Months**	
Mercy Health	
CF-I6 catheter	14.6 (10.3 to 19.0)
CF-I56 catheter	19.8 (14.8 to 24.8)
Difference	−5.2 (−12.4 to 2.0)
Mayo Clinic	
CF-I6 catheter	11.9 (8.7 to 15.1)
CF-I56 catheter	8.8 (1.0 to 16.6)
Difference	3.1 (−5.5 to 11.7)
Mean difference across Mercy catheter and Mayo Clinic	−1.8 (−7.3 to 3.7)

Abbreviations: CF-I6, catheter with contact force and 6-hole irrigation; CF-I56, catheter with contact force and 56-hole irrigation.

### Sensitivity Analyses

After the removal of hospital size (number of beds) as a balancing covariate at Mayo Clinic, the risk of safety outcomes was consistent with the primary results (−3.3%; 90% CI, −8.9% to 2.1%) (eTable 5 in the Supplement). Similarly, among patients who had been prescribed a class I or III antiarrhythmic medication in the 6 months before ablation, the risk of safety outcome was consistent with the primary results (−1.7%; 90% CI, −5.7% to 2.2%) (eTable 6 in the Supplement).

## Discussion

With the use of a distributed analytic model leveraging EHR data at 2 large health care systems, the CF-I6 catheter met the prespecified noninferiority safety criterion for persistent AF ablation compared with the CF-I56 catheter. Effectiveness outcomes were similar at 12 months. These results provide much-needed data about the safety and effectiveness of the CF-I6 catheter, which has been used during usual clinical practice for persistent AF ablation, despite lacking FDA approval for this condition. Our study also demonstrates the immense potential for using data from health information technology systems for medical device evaluations.

The clinical outcome results are reassuring about the safety of the CF-I6 catheter when used for ablation of persistent AF. The overall primary safety event rate was less than 7% at both health care systems. In the PRECEPT (Prospective Review of the Safety and Effectiveness of the ThermoCool SmartTouch SF Catheter Evaluated for Treating Symptomatic Persistent AF) trial used to support FDA approval of the CF-I56 catheter for persistent AF, the rate of adverse events in a similar composite safety end point was close to the current study (approximately 4%).^18^ The overall safety and effectiveness of the CF-I6 catheter are not surprising because procedural approaches are often similar between the catheters and between ablation for paroxysmal and persistent AF, although there may be heterogeneity in adoption of ablation catheters across different institutions, which may affect the generalizability of the findings.

In addition to the clinical findings, our study demonstrates the success of using both health care system supply chain data (for device identification) and EHR data for a medical device evaluation and can serve as a prototype for comparative studies using electronic health care databases for regulatory decisions. In 2021, the FDA released a report^32^ describing 90 examples of use of evidence from routine clinical practice to inform regulatory decision-making; only 3 used medical records for premarket approval devices, and none used EHR data as the sole data source. Most examples using medical records were for label expansions of devices cleared through the 510(k) and the de novo pathway, which do not have evidentiary standards as strong as the requirements for premarket approval devices.^33,34^

This study is the first retrospective cohort study using data from EHRs and health system supply chain data as the sole sources of clinical evidence for a medical device indication extension application. Nearly all prior studies for label extension have relied on case report forms, which require significant manual effort. However, we leveraged structured data to define computable phenotypes for patient populations, covariates, and effectiveness outcomes. Medical record review was only performed after applying a sensitive algorithm to identify patient records that might contain safety events, thus obviating the need to review medical records of all 1450 study patients. All steps were performed through standardized data extraction across 2 health care systems using the OMOP common data model to facilitate common analyses through standard queries in the 2 data sets; these distributed analytics allowed each health care system to maintain patient data behind organizational firewalls, with only final results combined. This approach demonstrates the scalability and efficiency of using fit-for-purpose health care system data for medical device safety and effectiveness evaluations. As we did, use of EHR data should include preanalysis protocol registration and transparent reporting of study findings.^35,36^

### Limitations

Our study should be considered in the context of its limitations. First, we ascertained safety outcomes through medical record review using a sensitive medical record identification algorithm, but we did not examine the negative predictive value, sensitivity, or specificity of our algorithm because this would have required assessments for 14 components of the composite safety outcome that have a low incidence. Therefore, because we did not examine these aspects of algorithm performance, it is possible that some patients had safety events that were not identified. However, we used multiple approaches to improve the sensitivity of the initial identification of medical records that contained potential safety end points and reviewed approximately one-sixth of all medical records. Furthermore, we would not expect the algorithm’s accuracy to differ between the 2 catheters of interest. Finally, given the seriousness of most of the safety events, it is unlikely that our criteria, which emphasized hospitalizations, would have missed many such outcomes. Second, we did not perform medical record review for effectiveness outcomes; however, we developed algorithms with acceptable positive predictive value for these events. Third, it is possible that there was incomplete ascertainment of some outcomes if patients did not follow up at Mercy Health or Mayo Clinic. Fourth, we did not validate our approach for identifying persistent AF. However, we used codes for patients as provided by clinicians in clinical practice. Fifth, as with any observational study, our findings may be limited by the presence of residual confounding, and although we used robust observational methods, additional techniques, such as falsification testing, could provide reassurance against residual confounding.

## Conclusions

This cohort study found that the rate of a composite safety end point was noninferior and 12-month effectiveness was similar among patients treated with the CF-I6 catheter compared with patients treated with the CF-I56 catheter for persistent AF ablation in 2 health care systems. These results suggest the potential to leverage health system data for safety and effectiveness evaluations of medical devices.

## Notes

Supplement. **eTable 1.** Patient Follow-up 365 Days After Index Catheter Ablation With a Catheter with Contact Force and 6-hole Irrigation or a Catheter with Contact Force and 56-hole Irrigation **eTable 2.** Absolute Standardized Differences (ASDs) According to Unadjusted (Crude) Data and Different Propensity Score Methods Among Patients Undergoing Ablation for Persistent Atrial Fibrillation in the Mercy Database **eTable 3.** Absolute Standardized Differences (ASDs) According to Unadjusted (Crude) Data and Different Propensity Score Methods Among Patients Undergoing Ablation for Persistent Atrial Fibrillation in the Mayo Clinic Database **eTable 4.** Summary of Charts Reviewed for Safety Outcome **eTable 5.** Cumulative Incidences and Risk Differences of the Primary Composite Safety Outcome Among Patients Undergoing Ablation for Persistent Atrial Fibrillation Using Propensity Score Balanced Data With Exclusion of Hospital Bed Size From the Propensity Score Model in the Mayo Clinic Database **eTable 6.** Cumulative Incidences and Risk Differences of the Primary Composite Safety Outcome Among Patients Undergoing Ablation for Persistent Atrial Fibrillation With a Prior Prescription of Class I or III Anti-arrhythmic Drug Within the 6 Months Before the Index Ablation Using Propensity Score Balanced Data **eFigure 1.** Cumulative Incidence of Primary Safety Outcome Among Covariate-Balanced Data at Mercy **eFigure 2.** Cumulative Incidence of Primary Safety Outcome Among Covariate-Balanced Data at Mayo Clinic **eFigure 3.** Cumulative Incidence of Exploratory Effectiveness Outcome at 365 Days Among Covariate-Balanced Data at Mercy **eFigure 4.** Cumulative Incidence of Exploratory Effectiveness Outcome at 365 Days Among Covariate-Balanced Data at Mayo Clinic
