# Supplementary material for: Safety and Effectiveness of a Catheter With Contact Force and 6-Hole Irrigation for Ablation of Persistent Atrial Fibrillation in Routine Clinical Practice
Source: JAMA Netw Open. 2022 Aug 17;5(8):e2227134. doi: 10.1001/jamanetworkopen.2022.27134 (PMC9386540; doi:10.1001/jamanetworkopen.2022.27134)
Supplement: Supplement. — eTable 1. Patient Follow-up 365 Days After Index Catheter Ablation With a Catheter with Contact Force and 6-hole Irrigation or a Catheter with Contact Force and 56-hole Irrigation eTable 2. Absolute Standardized Differences (ASDs) According to Unadjusted (Crude) Data and Different Propensity Score Methods Among Patients Undergoing Ablation for Persistent Atrial Fibrillation in the Mercy Database eTable 3. Absolute Standardized Differences (ASDs) According to Unadjusted (Crude) Data and Different Propensity Score Methods Among Patients Undergoing Ablation for Persistent Atrial Fibrillation in the Mayo Clinic Database eTable 4. Summary of Charts Reviewed for Safety Outcome eTable 5. Cumulative Incidences and Risk Differences of the Primary Composite Safety Outcome Among Patients Undergoing Ablation for Persistent Atrial Fibrillation Using Propensity Score Balanced Data With Exclusion of Hospital Bed Size From the Propensity Score Model in the Mayo Clinic Database eTable 6. Cumulative Incidences and Risk Differences of the Primary Composite Safety Outcome Among Patients Undergoing Ablation for Persistent Atrial Fibrillation With a Prior Prescription of Class I or III Anti-arrhythmic Drug Within the 6 Months Before the Index Ablation Using Propensity Score Balanced Data eFigure 1. Cumulative Incidence of Primary Safety Outcome Among Covariate-Balanced Data at Mercy eFigure 2. Cumulative Incidence of Primary Safety Outcome Among Covariate-Balanced Data at Mayo Clinic eFigure 3. Cumulative Incidence of Exploratory Effectiveness Outcome at 365 Days Among Covariate-Balanced Data at Mercy eFigure 4. Cumulative Incidence of Exploratory Effectiveness Outcome at 365 Days Among Covariate-Balanced Data at Mayo Clinic [file jamanetwopen-e2227134-s001.pdf]

Supplementary Online Content

Dhruva SS, Zhang S, Chen J, et al. Safety and effectiveness of a catheter with contact force and 6-hole irrigation for ablation of persistent atrial fibrillation in routine clinical practice. *JAMA Netw Open*. 2022;5(8):e2227134. doi:10.1001/jamanetworkopen.2022.27134

eTable 1. Patient Follow-up 365 Days After Index Catheter Ablation With a Catheter with Contact Force and 6-hole Irrigation or a Catheter with Contact Force and 56-hole Irrigation

eTable 2. Absolute Standardized Differences (ASDs) According to Unadjusted (Crude) Data and Different Propensity Score Methods Among Patients Undergoing Ablation for Persistent Atrial Fibrillation in the Mercy Database

eTable 3. Absolute Standardized Differences (ASDs) According to Unadjusted (Crude) Data and Different Propensity Score Methods Among Patients Undergoing Ablation for Persistent Atrial Fibrillation in the Mayo Clinic Database

eTable 4. Summary of Charts Reviewed for Safety Outcome

eTable 5. Cumulative Incidences and Risk Differences of the Primary Composite Safety Outcome Among Patients Undergoing Ablation for Persistent Atrial Fibrillation Using Propensity Score Balanced Data With Exclusion of Hospital Bed Size From the Propensity Score Model in the Mayo Clinic Database

eTable 6. Cumulative Incidences and Risk Differences of the Primary Composite Safety Outcome Among Patients Undergoing Ablation for Persistent Atrial Fibrillation With a Prior Prescription of Class I or III Anti-arrhythmic Drug Within the 6 Months Before the Index Ablation Using Propensity Score Balanced Data

eFigure 1. Cumulative Incidence of Primary Safety Outcome Among Covariate-Balanced Data at Mercy

eFigure 2. Cumulative Incidence of Primary Safety Outcome Among Covariate-Balanced Data at Mayo Clinic

eFigure 3. Cumulative Incidence of Exploratory Effectiveness Outcome at 365 Days Among Covariate-Balanced Data at Mercy

eFigure 4. Cumulative Incidence of Exploratory Effectiveness Outcome at 365 Days Among Covariate-Balanced Data at Mayo Clinic

This supplementary material has been provided by the authors to give readers additional information about their work.

© 2022 Dhruva SS et al. *JAMA Network Open.*

eTable 1. Patient Follow-up 365 Days After Index Catheter Ablation With a Catheter with Contact Force and 6-hole Irrigation or a Catheter with Contact Force and 56-hole Irrigation STSF Catheters

	Mercy		Mayo Clinic	
	**CF-I6**	**CF-I56**	**CF-I6**	**CF-I56**
	N (%)	N (%)	N (%)	N (%)
**Patients who met all eligibility criteria**	**186**	**763**	**337**	**164**
Incomplete follow-up for 1-year effectiveness outcomes	13 (7.0)	299 (39.2)	97 (28.8)	63 (38.4)
Death within 365 days from index procedure	1 (0.5)	8 (1.0)	0 (0.0)	0 (0.0)
Administrative censoring (Data Extract Date-Index Date < 365 days)	1 (0.5)	258 (33.8)	74 (22.0)	60 (36.6%)
Patient lost to follow-up (last health care encounter documented within 365 days from index procedure)	11 (5.9)	33 (4.3)	23 (6.8)	3 (1.8%)

Abbreviations:

CF-I6, catheter with contact force and 6-hole irrigation

CF-I56, catheter with contact force and 56-hole irrigation

© 2022 Dhruva SS et al. *JAMA Network Open.*

eTable 2. Absolute standardized differences (ASDs) According to Unadjusted (Crude) Data and Different Propensity Score Methods among Patients Undergoing Ablation for Persistent Atrial Fibrillation in the Mercy Database

Characteristic	CF-I56 (n=763)	CF-I6 (n=186)	Crude (unadjusted) ASD	Stratification ASD	Untrimmed Weighting ASD	Weights Trimmed at the 99th percentile ASD	Weights Trimmed at the 95th percentile ASD	Weights Trimmed at the 90th percentile ASD
Age, years			0.110	0.334	0.031	0.038	0.069	0.083
<65	349 (45.7%)	75 (40.3%)						
65-74	310 (40.6%)	83 (44.6%)						
≥75	104 (13.6%)	28 (15.1%)						
Sex			0.054	0.248	0.008	0.042	0.099	0.108
Male	544 (71.3%)	128 (68.8%)						
Female	219 (28.7%)	58 (31.2%)						
Race			0.038	0.203	0.016	0.010	0.018	0.017
White	732 (95.9%)	177 (95.2%)						
Other[a]	31 (4.1%)	9 (4.8%)						
Elixhauser comorbidity index			0.067	0.210	0.036	0.021	0.023	0.024
≤3	183 (24.0%)	50 (26.9%)						
>3	580 (76.0%)	136 (73.1%)						
Index year			1.991	0.627	0.017	0.024	0.059	0.069
2014-2018	184 (24.1%)	174 (93.5%)						
2019-2021	579 (75.9%)	12 (6.5%)						
Body mass index, kg/m^2	32.8 (6.7)	31.8 (5.8)	0.158	0.155	0.031	0.034	0.001	0.009
Body mass index missing	0 (0%)	0 (0%)						
Percutaneous coronary intervention	49 (6.4%)	13 (7.0%)	0.023	0.313	0.043	0.025	0.001	0.003
Coronary artery bypass grafting	67 (8.8%)	13 (7.0%)	0.067	0.182	0.003	0.015	0.013	0.009
Hypertension	615 (80.6%)	147 (79.0%)	0.039	0.345	0.057	0.058	0.044	0.045
Diabetes mellitus	198 (26.0%)	38 (20.4%)	0.131	0.362	0.010	0.015	0.009	0.006
Obstructive sleep apnea	364 (47.7%)	86 (46.2%)	0.029	0.681	0.017	0.006	0.035	0.040
Congestive heart failure	318 (41.7%)	77 (41.4%)	0.006	0.272	0.001	0.000	0.005	0.006
Chronic pulmonary disease	215 (28.2%)	52 (28.0%)	0.005	0.130	0.007	0.007	0.023	0.027
Stroke or transient ischemic attack or thromboembolism	96 (12.6%)	20 (10.8%)	0.057	0.268	0.002	0.014	0.008	0.007
Chronic renal disease	127 (16.6%)	29 (15.6%)	0.029	0.068	0.018	0.007	0.009	0.008
Anemia	62 (8.1%)	12 (6.5%)	0.064	0.230	0.003	0.004	0.023	0.024

© 2022 Dhruva SS et al. *JAMA Network Open.*

Characteristic	CF-I56 (n=763)	CF-I6 (n=186)	Crude (unadjusted) ASD	Stratification ASD	Untrimmed Weighting ASD	Weights Trimmed at the 99th percentile ASD	Weights Trimmed at the 95th percentile ASD	Weights Trimmed at the 90th percentile ASD
Valve replacement	27 (3.5%)	8 (4.3%)	0.039	0.171	0.013	0.005	0.013	0.019
Mitral valve stenosis	5 (0.7%)	3 (1.6%)	0.091	0.114	0.043	0.045	0.012	0.019
Vascular disease	274 (35.9%)	58 (31.2%)	0.100	0.095	0.029	0.026	0.011	0.008
Hospitalizations: atrial fibrillation related	315 (41.3%)	89 (47.8%)	0.132	0.290	0.050	0.067	0.122	0.132
Electrical cardioversion for atrial fibrillation	543 (71.2%)	136 (73.1%)	0.044	0.116	0.008	0.017	0.017	0.018
Supraventricular arrythmia	352 (46.1%)	89 (47.8%)	0.034	0.339	0.035	0.031	0.038	0.042
Ventricular arrhythmia	54 (7.1%)	17 (9.1%)	0.076	0.107	0.066	0.032	0.014	0.009
Implantable cardioverter defibrillator or pacemaker	61 (8.0%)	18 (9.7%)	0.059	0.316	0.017	0.026	0.056	0.060
Class I or III antiarrhythmic drugs	438 (57.4%)	112 (60.2%)	0.057	0.120	0.040	0.054	0.087	0.094
Class II or IV antiarrhythmic drugs	697 (91.3%)	172 (92.5%)	0.041	0.121	0.009	0.001	0.035	0.045
Anticoagulants	681 (89.3%)	167 (89.8%)	0.017	0.153	0.014	0.005	0.029	0.039
Antiplatelets	618 (81.0%)	155 (83.3%)	0.061	0.298	0.019	0.007	0.015	0.020
Operator volume in the 12 months prior to the index procedure			0.759	0.589	0.245	0.272	0.277	0.276
<25	247 (33.1%[b])	9 (4.9% [b])						
25-50	49 (6.6% [b])	18 (9.9% [b])						
>50	450 (60.3% [b])	155 (85.2% [b])						
Missing	17 (2.2%)	4 (2.2%)						
Hospital bed size			-	-	-	-	-	-
≥500	763 (100%)	186 (100%)						
<500	0 (0%)	0 (0%)						
**Number of ASDs > 0.20**			**2**	**17**	**1**	**1**	**1**	**1**
**Mean ASD**			**0.15**	**0.26**	**0.03**	**0.03**	**0.04**	**0.04**

[a] "Other" refers to American Indian or Alaska Native, Asian, Black or African American, Hispanic or Latino, Native Hawaiian or Other Pacific Islander, Other, and Unknown

[b] Percentage based on data with known values.

ASD: absolute standardized difference

© 2022 Dhruva SS et al. *JAMA Network Open.*

eTable 3. Absolute standardized differences (ASDs) According to Unadjusted (Crude) Data and Different Propensity Score Methods among Patients Undergoing Ablation for Persistent Atrial Fibrillation in the Mayo Clinic Database

Characteristic	CF-I56 (n=164)	CF-I6 (n=337)	Crude (unadjusted) ASD	Stratification ASD	Untrimmed Weighting ASD	Weights Trimmed at the 99th percentile ASD	Weights Trimmed at the 95th percentile ASD	Weights Trimmed at the 90th percentile ASD
Age, years			0.279	1.407	0.505	0.151	0.177	0.177
<65	68 (41.5%)	186 (55.2%)						
65-74	71 (43.3%)	114 (33.8%)						
≥75	25 (15.2%)	37 (11.0%)						
Sex			0.010	0.642	0.287	0.169	0.061	0.061
Male	118 (72.0%)	244 (72.4%)						
Female	46 (28.0%)	93 (27.6%)						
Race			0.138	0.189	0.248	0.138	0.005	0.005
White	162 (98.8%)	326 (96.7%)						
Other[a]	2 (1.2%)	11 (3.3%)						
Elixhauser comorbidity index			0.267	0.428	0.006	0.189	0.193	0.193
≤3	39 (23.8%)	121 (35.9%)						
>3	125 (76.2%)	216 (64.1%)						
Index year			0.613	0.603	1.032	0.102	0.115	0.115
2014-2018	42 (25.6%)	183 (54.3%)						
2019-2021	122 (74.4%)	154 (45.7%)						
Body mass index, kg/m^2	32.9 (7.8)	32.3 (6.4)	0.086	0.250	0.024	0.253	0.038	0.038
Body mass index missing	1 (0.6%)	11 (3.3%)	0.193	0.205	0.250	0.155	0.028	0.028
Percutaneous coronary intervention	4 (2.4%)	7 (2.1%)	0.024	0.210	0.203	0.170	0.117	0.117
Coronary artery bypass grafting	4 (2.4%)	7 (2.1%)	0.024	0.172	0.186	0.032	0.077	0.077
Hypertension	116 (70.7%)	199 (59.1%)	0.247	0.890	0.999	0.253	0.199	0.199
Diabetes mellitus	37 (22.6%)	47 (13.9%)	0.224	0.411	0.459	0.197	0.210	0.210
Obstructive sleep apnea	75 (45.7%)	136 (40.4%)	0.109	0.931	1.275	0.142	0.064	0.064
Congestive heart failure	84 (51.2%)	138 (40.9%)	0.207	0.645	0.341	0.191	0.077	0.077
Chronic pulmonary disease	48 (29.3%)	73 (21.7%)	0.175	0.660	0.746	0.123	0.118	0.118
Stroke or transient ischemic attack or thromboembolism	23 (14.0%)	28 (8.3%)	0.182	1.558	0.107	0.258	0.097	0.097
Chronic renal disease	23 (14.0%)	19 (5.6%)	0.285	1.626	0.016	0.259	0.198	0.198
Anemia	14 (8.5%)	21 (6.2%)	0.088	0.318	0.338	0.116	0.083	0.083

© 2022 Dhruva SS et al. *JAMA Network Open.*

Characteristic	CF-I56 (n=164)	CF-I6 (n=337)	Crude (unadjusted) ASD	Stratification ASD	Untrimmed Weighting ASD	Weights Trimmed at the 99th percentile ASD	Weights Trimmed at the 95th percentile ASD	Weights Trimmed at the 90th percentile ASD
Valve replacement	6 (3.7%)	14 (4.2%)	0.026	0.260	0.283	0.173	0.024	0.024
Mitral valve stenosis	1 (0.6%)	5 (1.5%)	0.086	0.199	0.173	0.170	0.163	0.163
Vascular disease	64 (39.0%)	178 (52.8%)	0.280	0.882	0.082	0.251	0.213	0.213
Hospitalizations: atrial fibrillation related	86 (52.4%)	131 (38.9%)	0.275	0.597	0.482	0.322	0.162	0.162
Electrical cardioversion for atrial fibrillation	111 (67.7%)	139 (41.2%)	0.551	0.549	0.351	0.377	0.458	0.458
Supraventricular arrythmia	105 (64.0%)	188 (55.8%)	0.169	0.604	0.893	0.210	0.101	0.101
Ventricular arrhythmia	24 (14.6%)	38 (11.3%)	0.100	0.525	1.036	0.040	0.004	0.004
Implantable cardioverter defibrillator or pacemaker	15 (9.1%)	23 (6.8%)	0.086	0.356	0.372	0.268	0.115	0.115
Class I or III antiarrhythmic drugs	93 (56.7%)	155 (46.0%)	0.216	0.683	0.083	0.284	0.173	0.173
Class II or IV antiarrhythmic drugs	136 (82.9%)	274 (81.3%)	0.042	0.592	0.657	0.459	0.183	0.183
Anticoagulants	126 (76.8%)	205 (60.8%)	0.351	0.891	1.074	0.581	0.318	0.318
Antiplatelets	46 (28.0%)	88 (26.1%)	0.044	0.935	0.715	0.071	0.031	0.031
Operator volume in the 12 months prior to the index procedure			0.548	1.506	1.360	0.059	0.257	0.257
<25	23 (14.0%)	95 (28.2%)						
25-50	135 (82.3%)	198 (58.8%)						
>50	6 (3.7%)	44 (13.1%)						
Missing	0 (%)	0 (%)						
Hospital bed size			3.012	0.324	0.339	0.479	1.599	1.599
≥500	10 (6.1%)	301 (89.3%)						
<500	154 (93.9%)	36 (10.7%)						
**Number of ASDs > 0.20**			**14**	**28**	**23**	**13**	**6**	**6**
**Mean ASD**			**0.29**	**0.65**	**0.48**	**0.21**	**0.18**	**0.18**

[a] "Other" refers to American Indian or Alaska Native, Asian, Black or African American, Hispanic or Latino, Native Hawaiian or Other Pacific Islander, Other, and Unknown

ASD: absolute standardized difference

Abbreviations:

CF-I6, catheter with contact force and 6-hole irrigation

CF-I56, catheter with contact force and 56-hole irrigation

© 2022 Dhruva SS et al. *JAMA Network Open.*

eTable 4. Summary of Charts Reviewed for Safety Outcome

	Mercy (N=949)	Mayo Clinic (N=501)
**Total number of patients who met at least one of the 3 criteria for physician chart review**	77 (8.1%)	158 (31.5%)
**Readmission within 7 days after ablation**	19 (24.7%)	23 (14.6%)
**Emergency department visit within 7 days after ablation**	23 (29.9%)	26 (16.5%)
**Index ablation length of stay ≥ 48 hours**	32 (41.6%)	75 (47.5%)
**With a diagnosis or procedure code of safety events of interest that may not be detected by the above criteria**	12 (15.6%)	67 (42.4%)

Note: Because some patients met multiple criteria, their charts were identified for chart review using multiple approaches. The denominator for the total number of patients who met at least one criterion for physician chart review was the total number of patients within each health system. The denominator for subsequent rows is the total number of patients who met at least one of the criteria for physician chart review.

© 2022 Dhruva SS et al. *JAMA Network Open.*

eTable 5. Cumulative Incidences and Risk Differences of the Primary Composite Safety Outcome among Patients Undergoing Ablation for Persistent Atrial Fibrillation Using Propensity Score Balanced Data with Exclusion of Hospital Bed Size from the Propensity Score Model in the Mayo Clinic Database

Study Group	Adjusted Cumulative Incidence (90% CI)	Weight (Inverse of Variance)
**Mayo Clinic**		
CF-I6	6.2% (4.0% to 8.4%)	-
CF-I56	9.6% (4.5% to 14.6%)	-
**Difference**	-3.3% (-8.9% to 2.1%)	879.12

[a] The average treatment effect on the treated weights with weights trimmed at the 95th percentile of the weight distribution in the comparator group. The weight column denotes the weight given to the data source in the meta-analysis, based on the inverse of the variance of the device effect.

Abbreviations:

CF-I6, catheter with contact force and 6-hole irrigation

CF-I56, catheter with contact force and 56-hole irrigation

© 2022 Dhruva SS et al. *JAMA Network Open.*

eTable 6. Cumulative Incidences and Risk Differences of the Primary Composite Safety Outcome among Patients Undergoing Ablation for Persistent Atrial Fibrillation with a Prior Prescription of Class I or III Anti-arrhythmic Drug within the 6 Months Before the Index Ablation using Propensity Score Balanced Data[a]

Study Group	Adjusted Cumulative Incidence (90% CI)	Weight (Inverse of Variance)
**Mercy**		
CF-I6	3.6% (0.7% to 6.5%)	-
CF-I56	4.3% (0.8% to 7.7%)	-
**Difference**	-0.7% (-5.3% to 3.9%)	1275.91
**Mayo Clinic**		
CF-I6	4.5% (1.7% to 7.2%)	-
CF-I56	9.1% (2.0% to 16.1%)	-
**Difference**	-4.5% (-12.1% to 3.0%)	466.43
**Average Difference across Mercy and Mayo Clinic**	-1.7% (-5.7% to 2.2%)	-

[a] The average treatment effect on the treated weights with weights trimmed at the 95th percentile of the weight distribution in the comparator group. The weight column denotes the weight given to the healthcare system data source in the meta-analysis based on the inverse of the variance of the device effect, which accounts for the sample size in the Mercy and Mayo Clinical study population.

Abbreviations:

CF-I6, catheter with contact force and 6-hole irrigation

CF-I56, catheter with contact force and 56-hole irrigation

© 2022 Dhruva SS et al. *JAMA Network Open.*

eFigure 1. Cumulative Incidence of Primary Safety Outcome Among Covariate-Balanced Data at Mercy

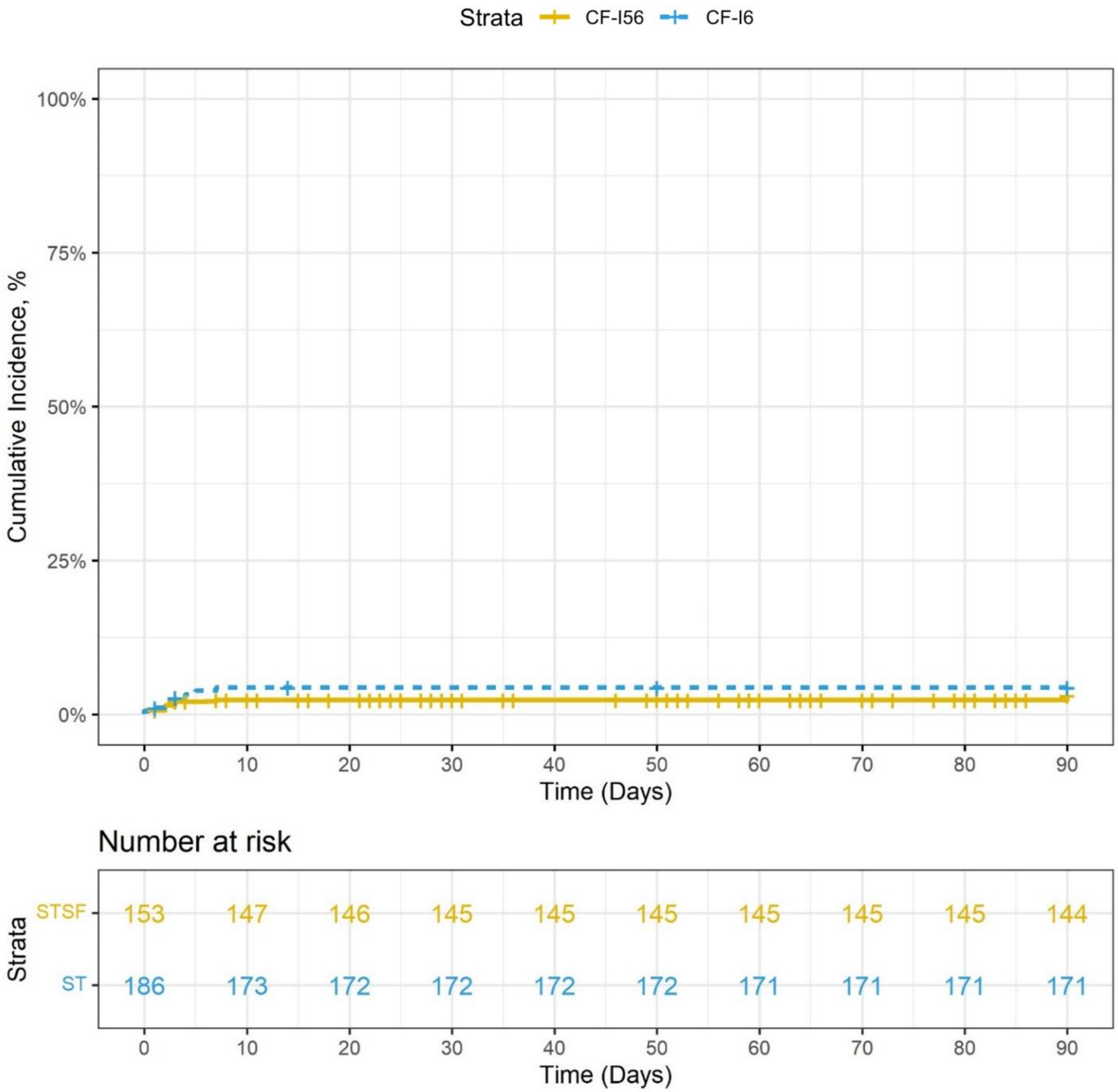

© 2022 Dhruva SS et al. *JAMA Network Open.*

eFigure 2. Cumulative Incidence of Primary Safety Outcome Among Covariate-Balanced Data at Mayo Clinic

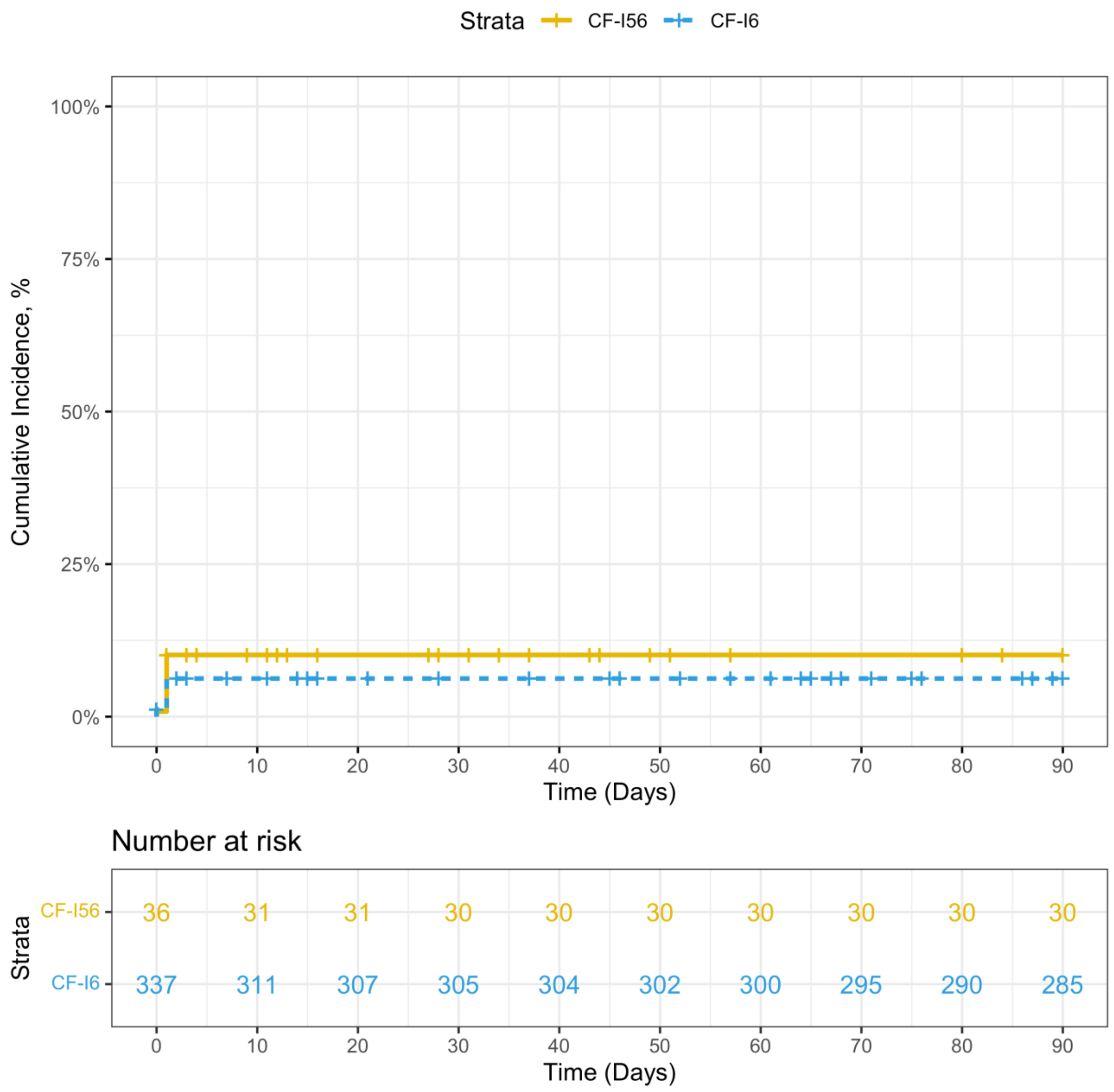

© 2022 Dhruva SS et al. *JAMA Network Open.*

eFigure 3. Cumulative Incidence of Exploratory Effectiveness Outcome at 365 Days Among Covariate-Balanced Data at Mercy

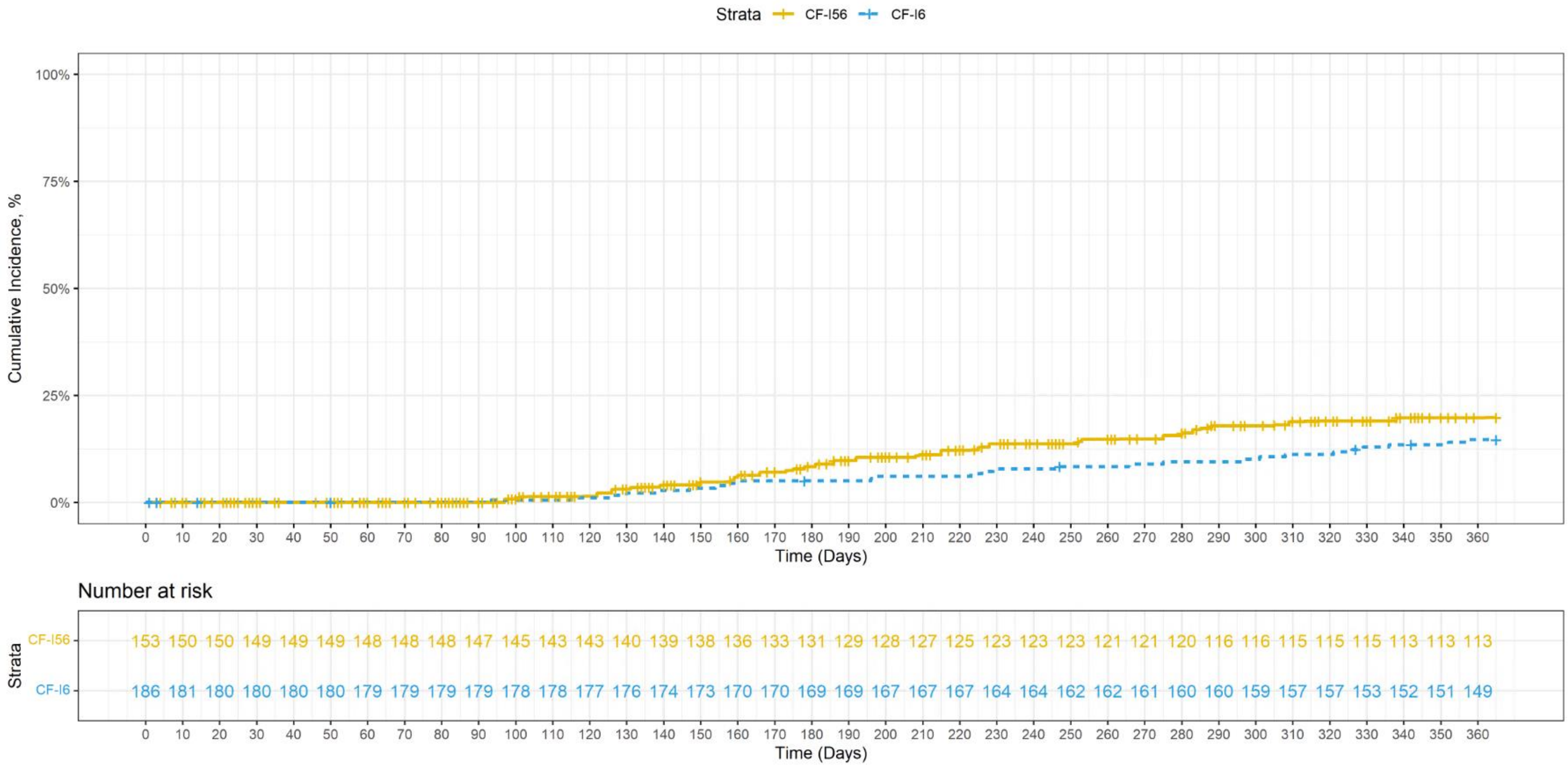

© 2022 Dhruva SS et al. *JAMA Network Open.*

eFigure 4. Cumulative Incidence of Exploratory Effectiveness Outcome at 365 Days Among Covariate-Balanced Data at Mayo Clinic

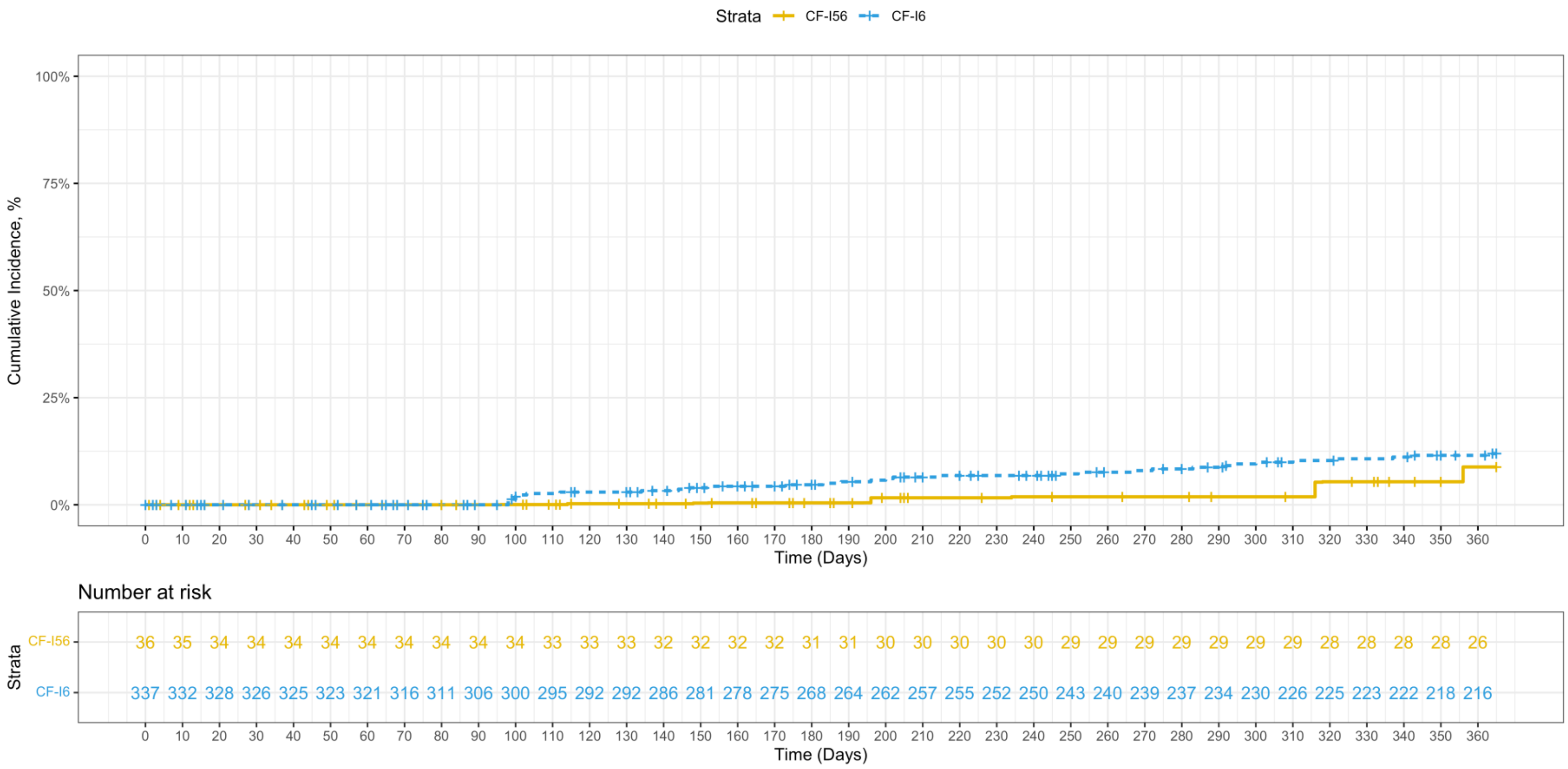

© 2022 Dhruva SS et al. *JAMA Network Open.*
